# Optimizing 3DCT image registration for interfractional changes in carbon-ion prostate radiotherapy

**DOI:** 10.1038/s41598-023-34339-w

**Published:** 2023-05-08

**Authors:** Ryusuke Hirai, Shinichiro Mori, Hiroki Suyari, Hiroshi Tsuji, Hitoshi Ishikawa

**Affiliations:** 1 National Institutes for Quantum Science and Technology, Quantum Life and Medical Science Directorate, Institute for Quantum Medical Science, Inage-ku, Chiba, 263-8555 Japan; 2 Corporate Research and Development Center, Toshiba Corporation, Kanagawa, 212-8582 Japan; 3 Department of Information and Image Sciences, Faculty of Engineering, Chiba University, Inage-ku, Chiba, 263-8522 Japan; 4 QST Hospital, National Institutes for Quantum Science and Technology, Inage-ku, Chiba, 263-8555 Japan

**Keywords:** Radiotherapy, Three-dimensional imaging

## Abstract

To perform setup procedures including both positional and dosimetric information, we developed a CT–CT rigid image registration algorithm utilizing water equivalent pathlength (WEPL)-based image registration and compared the resulting dose distribution with those of two other algorithms, intensity-based image registration and target-based image registration, in prostate cancer radiotherapy using the carbon-ion pencil beam scanning technique. We used the data of the carbon ion therapy planning CT and the four-weekly treatment CTs of 19 prostate cancer cases. Three CT–CT registration algorithms were used to register the treatment CTs to the planning CT. Intensity-based image registration uses CT voxel intensity information. Target-based image registration uses target position on the treatment CTs to register it to that on the planning CT. WEPL-based image registration registers the treatment CTs to the planning CT using WEPL values. Initial dose distributions were calculated using the planning CT with the lateral beam angles. The treatment plan parameters were optimized to administer the prescribed dose to the PTV on the planning CT. Weekly dose distributions using the three different algorithms were calculated by applying the treatment plan parameters to the weekly CT data. Dosimetry, including the dose received by 95% of the clinical target volume (CTV-D95), rectal volumes receiving > 20 Gy (RBE) (V20), > 30 Gy (RBE) (V30), and > 40 Gy (RBE) (V40), were calculated. Statistical significance was assessed using the Wilcoxon signed-rank test. Interfractional CTV displacement over all patients was 6.0 ± 2.7 mm (19.3 mm maximum standard amount). WEPL differences between the planning CT and the treatment CT were 1.2 ± 0.6 mm-H_2_O (< 3.9 mm-H_2_O), 1.7 ± 0.9 mm-H_2_O (< 5.7 mm-H_2_O) and 1.5 ± 0.7 mm-H_2_O (< 3.6 mm-H_2_O maxima) with the intensity-based image registration, target-based image registration, and WEPL-based image registration, respectively. For CTV coverage, the D95 values on the planning CT were > 95% of the prescribed dose in all cases. The mean CTV-D95 values were 95.8 ± 11.5% and 98.8 ± 1.7% with the intensity-based image registration and target-based image registration, respectively. The WEPL-based image registration was CTV-D95 to 99.0 ± 0.4% and rectal Dmax to 51.9 ± 1.9 Gy (RBE) compared to 49.4 ± 9.1 Gy (RBE) with intensity-based image registration and 52.2 ± 1.8 Gy (RBE) with target-based image registration. The WEPL-based image registration algorithm improved the target coverage from the other algorithms and reduced rectal dose from the target-based image registration, even though the magnitude of the interfractional variation was increased.

## Introduction

Particle beam treatment techniques have improved in the past few years, allowing a higher dose to the tumor with reduced doses to surrounding normal tissues. This has enabled the performance of dose escalation studies to increase the prescribed dose to the tumor^1^. Especially in carbon-ion pencil-beam scanning therapy, it has been possible to decrease the number of treatment fractions while increasing the dose (hypofractionated treatment) owing to this method’s unique physical and biological characteristics. Current treatment planning assumes a patient’s internal anatomy to be unchanged throughout the treatment course. However, recently, several reports using medical imaging have identified and quantified internal anatomical changes, and found changes in target shape occurring over time periods varying from hours to days (interfractional change)^2–4^.

The prostate as an example is generally influenced by intrafractional motion but does not undergo interfractional changes in shape^5,6^. Bowel and bladder filling can have an effect on internal anatomical changes, but can be partially controlled by drinking, and/or voiding schemes, rectal balloons, or the use of laxatives or enemas to control rectal filling^7–10^. These situations are commonly checked on the setup procedure, which registers the patient’s position to a reference position in the treatment room using digital radiography or CT. Image registration techniques have been integrated with the setup software to improve positional accuracy and throughput^11–13^. These techniques are frequently used in radiotherapy and aid oncologists, physicists, and therapists in targeting tumors accurately.

A widely used method of patient positional verification is the 2D-3D image registration technique, which uses a combination of 2D images and the volumetric CT data from treatment planning^14^. This technique calculates three translations and three rotations; however, it cannot provide tumor and organ geometrical information in 3D. Volumetric imaging of the patient is frequently performed in the treatment position with cone-beam CT (CBCT) in photon/particle beam treatment^15–17^. An alternative approach to CBCT is a CT scanner with the gantry on rails installed in the treatment room, which slides over the immobilized patient^18^. Any required couch translations and/or rotations are calculated by CT images acquired in the treatment room (treatment CT) and are compared to the planning CT. This CT–CT image registration is based on pixel intensity and does not include actual target positional information, and denser tissues such as bone show better registration than soft tissues. This means that the tumors located in soft tissues are not directly registered to their counterparts on the treatment planning CT. This allows tumors and/or surrounding tissues undergoing shape changes, to move out or into the treatment beam field, respectively^19–21^. After CT–CT image registration, the treatment couch is moved to adjust the tumor position so that it matches the tumor on the planning CT.

However, tumor and other anatomical sites were not completely registered to the reference position due to interfractional anatomical positional changes, the image registration with image pixel intensity and/or geometrical information was not fully satisfied to clinical staff to prevent interfractional dose degradations in the treatment. To solve this problem, it required the image registration including dosimetric information. Several researchers have introduced dosimetric assessment using CT–CT image registration of bony structures, fiducial markers, and target positions for intensity-modulated proton-beam^22^, regular proton-beam^23,24^, and carbon-ion beam^25–28^ therapy. These concepts essentially provide geometric information; radiologic pathlength variations, which are related to the charged particle beam stopping position at the distal surface of the target, may also be used for mapping of structures. Ideally, it is better to perform a full dosimetric analysis for the effect on interfractional beam penetration, however, it is still hard to use in clinic due to a bit longer computation time. Therefore, evaluating water equivalent pathlength (WEPL), rather than a full dosimetric analysis, would be efficient because of its short computation time^29–32^. The aim of this work is to develop and implement a WEPL based image registration algorithm and evaluate its performance using a cohort of 19 patients treated with C-ion therapy for prostate cancer.

## Results

### CTV displacement and volume variations

The CTV displacement, which was measured using the target displacement method, averaged over all patients was 6.0 ± 2.7 mm (mean ± SD). The largest CTV displacement through the treatment course was 11.9 ± 2.4 mm (no. 10) and the smallest one was 2.7 ± 1.1 mm (no. 1).

Regarding volume variations, the mean absolute error of the prostate volume difference was 0.96 ± 0.71 cc (max: 2.5 cc); the prostate volume was not significantly changed throughout the treatment course. The mean absolute bladder volume difference was 52.7 ± 38.44 cc and the maximum difference was 191.7 cc (no. 18). Three cases showed > 100 cc differences (no. 1, 5 and 18).

### WEPL variation with the image registration

**Figure 1:**
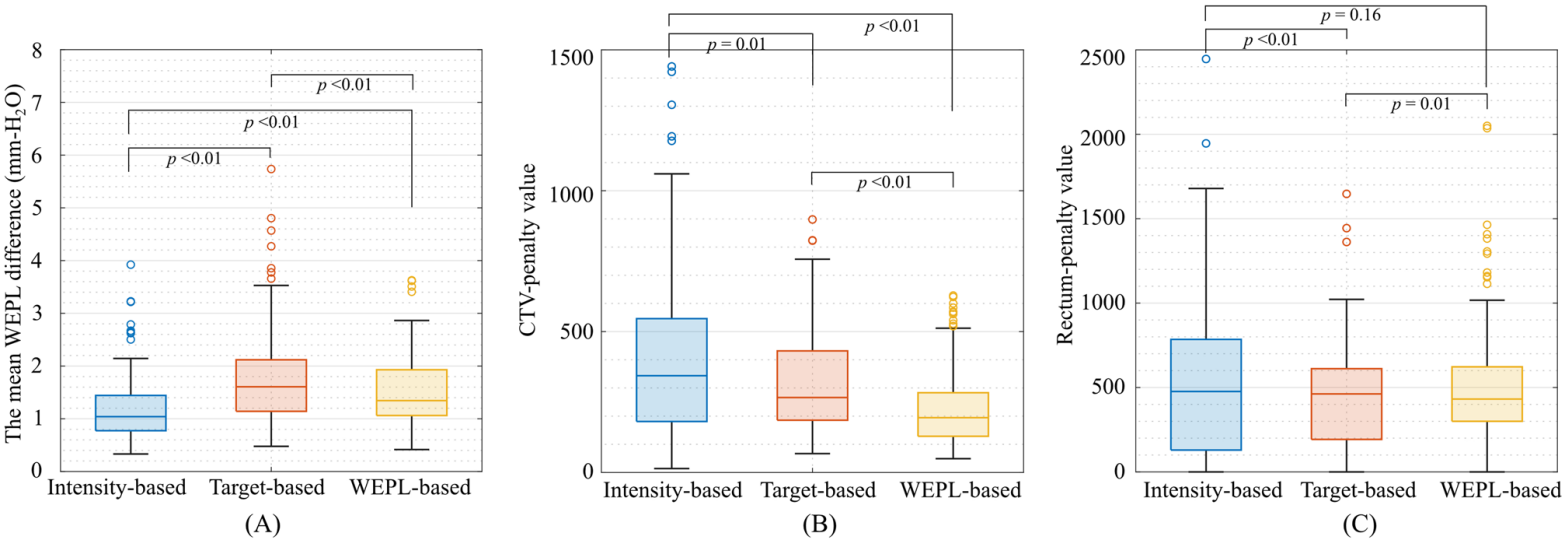
(**A**) WEPL differences with three image registration methods (intensity-based image registration, target-based image registration and WEPL-based image registration) over the PTV1. (**B**) The normalized CTV-penalty region outside the plan-PTV. (C) The normalized rectum-penalty region over the plan-PTV. The whiskers extend to the most extreme data points not considered outliers. An outlier (marked as a circle) is identified if it is > q3 + (q3 − q1) × 1.5 or < q1 − (q3 − q1) × 1.5. This image is created by MATLAB R2021a, (Mathworks, Natick MA, USA, https://www.mathworks.com).

WEPL differences from the intensity-based image registration, target-based image registration, and WEPL-based image registration are shown in Fig. 1A. The mean WEPL difference with the WEPL-based image registration was reduced to 1.5 ± 0.7 mm-H_2_O (mean ± SD) (< 3.6 mm-H_2_O) compared with those with the intensity-based image registration (= 1.2 ± 0.6 mm-H_2_O, < 3.9 mm-H_2_O) and the target-based image registration (= 1.7 ± 0.9 mm-H_2_O, < 5.7 mm-H_2_O).

The penalty values (CTV-penalty/rectum-penalty) averaged over all patients were 421.0 ± 332.9/576.7 ± 605.4, 322.0 ± 184.5/450.8 ± 331.8 and 231.0 ± 138.7/498.9 ± 357.9 with the intensity-based image registration, target-based image registration and WEPL-based image registration, respectively (Fig. 1B,C). WEPL-based image registration had fewer outlier values for the mean WEPL differences and CTV-penalty values compared to the intensity-based image registration and target-based image registration.

### Dose assessment

**Figure 2:**
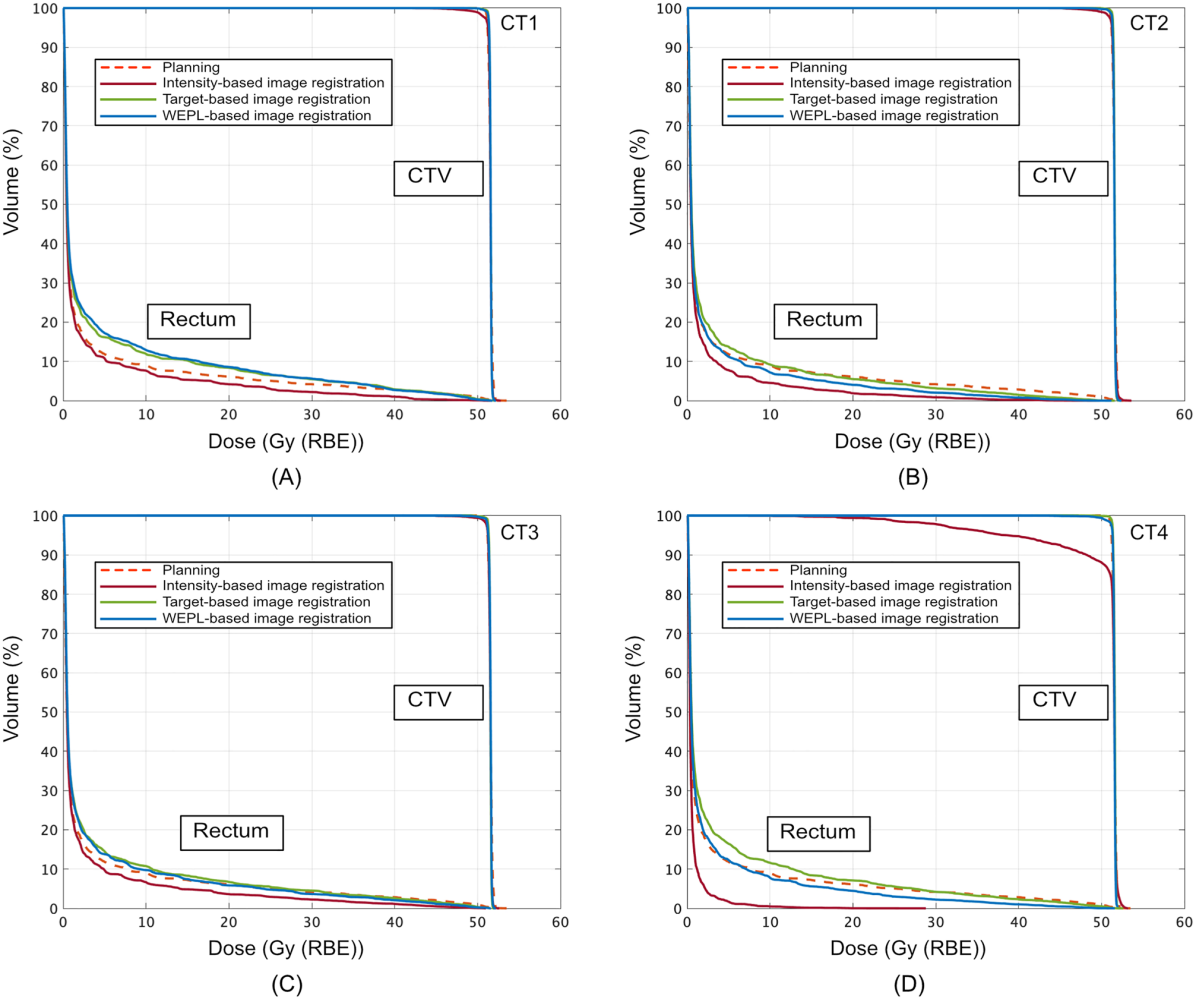
DVHs for the CTV and rectum on the planning CT and the treatment CTs ((**A**) CT1, (**B**) CT2, (**C**) CT3 and (**D**) CT4) with the intensity-based image registration, target-based image registration and WEPL-based image registration (case no. 7). This image is created by MATLAB R2021a, (Mathworks, Natick MA, USA, https://www.mathworks.com).

CTV displacement throughout the treatment course was measured; that for case no. 7 was 6.7 ± 2.0 mm, DVHs on the planning CT and the treatment CTs are shown in Fig. 2. All image registrations provided > 99% of the CTV-D95 on CT1–CT3 and had almost the same CTV-D95 value as the planning CT except the intensity-based image registration on CT4 (Fig. 2A–D). For CT4, intensity-based image registration degraded target coverage (CTV-D95 = 75.1%) (Fig. 2D). Rectal doses for the WEPL-based image registration were similar to those for the target-based image registration on CT1 and CT3. However, WEPL-based image registration decreased rectal doses compared to other two image registrations.

**Figure 3:**
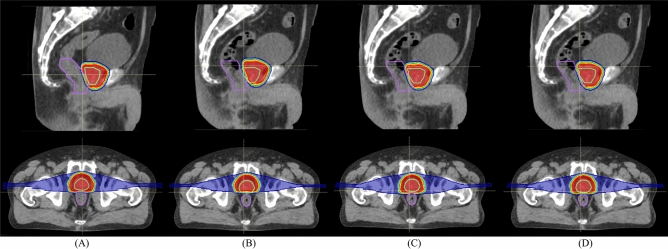
Dose distributions (case no.7) on (**A**) the planning CT and on the treatment CT (CT1) with (**B**) intensity-based image registration, (**C**) target-based image registration, and (**D**) WEPL-based image registration. Light green and purple lines outline the CTV and rectum, respectively.

To increase understanding of the situation in CT4, dose distributions on the planning CT and on the treatment CT (CT4) are shown in Fig. 3 (case no. 7). Dose distributions on the planning CT delivered a sufficient dose to the CTV (D95 = 99.2%) and reduced rectal dose (Dmax = 51.6 Gy (RBE)) (Fig. 3A), while dose distributions registered with the intensity-based image registration showed decreased target coverage due to the interfractional CTV positional variation (Fig. 3B). This could be affected by rectal filling variations. The posterior and apical aspects of the prostate were located out of the beam field on the CT1 with intensity-based image registration. Dose distributions with the intensity-based and target-based image registration did not take WEPL for target and rectum into account; as a result, degraded target dose and increased rectal dose were clearly identifiable on the axial sections compared to those on the planning CT (lower panel in Fig. 3B,C). On the other hand, the WEPL-based image registration registered the CTV within the beam field and satisfied target coverage and rectal dose reduction (Fig. 3D).

**Figure 4:**
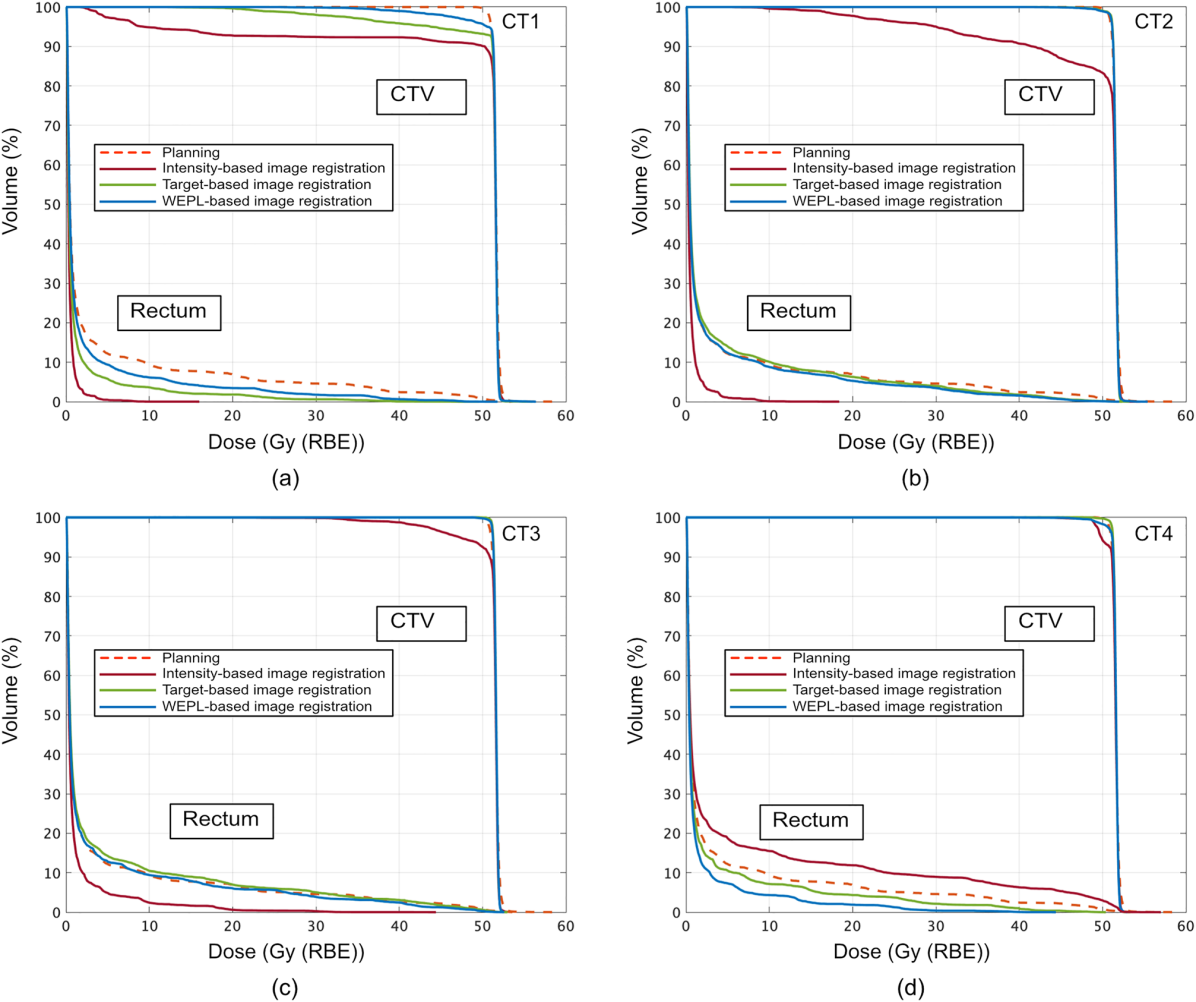
DVHs for the CTV and rectum on the planning CT and the treatment CTs ((**A**) CT1, (**B**) CT2, (**C**) CT3 and (**D**) CT4) with the intensity-based image registration, target-based image registration and WEPL-based image registration (case no. 2). This image is created by MATLAB R2021a, (Mathworks, Natick MA, USA, https://www.mathworks.com).

**Figure 5:**
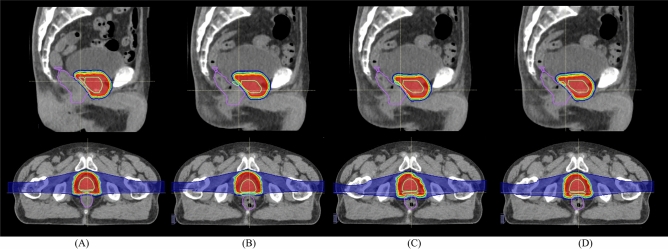
Dose distributions (case no.2) on (**A**) the planning CT and on the treatment CT (CT1) with (**B**) intensity-based image registration, (**C**) target-based image registration, and (**D**) WEPL-based image registration. Light green and purple lines outline the CTV and rectum, respectively.

Another case (case no.2) showed worst-dose degradation. CTV displacement throughout the treatment course was 9.5 ± 4.8 mm. Figures 4 and 5 shows DVHs and dose distributions on the planning (Figs. 4 and 5A) and the treatment CTs (Figs. 4 and 5B–D). For the intensity-based image registration, rectal doses on CT1-CT3 were lower than those with other image registrations, however, the CTV-D95 values were degraded to 18.1% on CT1, 58.3% on CT2, and 91.6% on CT3 (Fig. 4A–C). This is because CTV was out of the beam field (Fig. 5B). The CTV-95 value was improved to 96.6% on CT4, however, the rectal dose was increased (Fig. 4D). This registration was not satisfied with the target coverage (CTV-D95 = 66.1 ± 36.2%) and the rectal dose (Dmax = 15.8 ± 20.1 Gy (RBE)). The target-based image registration showed > 99% of the CTV-D95 (= 95.7 ± 7.2%) and rectal-Dmax [= 51.1 ± 1.7 Gy (RBE)], however, the CTV-D95 on CT1 was degraded to 84.9% (Fig. 4A), because CTV position was completely included within the beam field (Fig. 5C). This registration improved the results rather than those with the intensity-based image registration. While the WEPL image registration provided > 98% of the CTV-D95 on CT1-CT4 (= 98.9 ± 0.6%) and rectal-Dmax (= 50.1 ± 3.9%), it was similar result to that on the planning CT (CTV-D95 = 98.9%, rectal-Dmax = 53.8 Gy (RBE)). By considering WEL differences with penalty function, CTV position was included within the beam field (Fig. 5D). This registration satisfied with both target coverage and rectal dose rather than other registrations.

**Figure 6:**
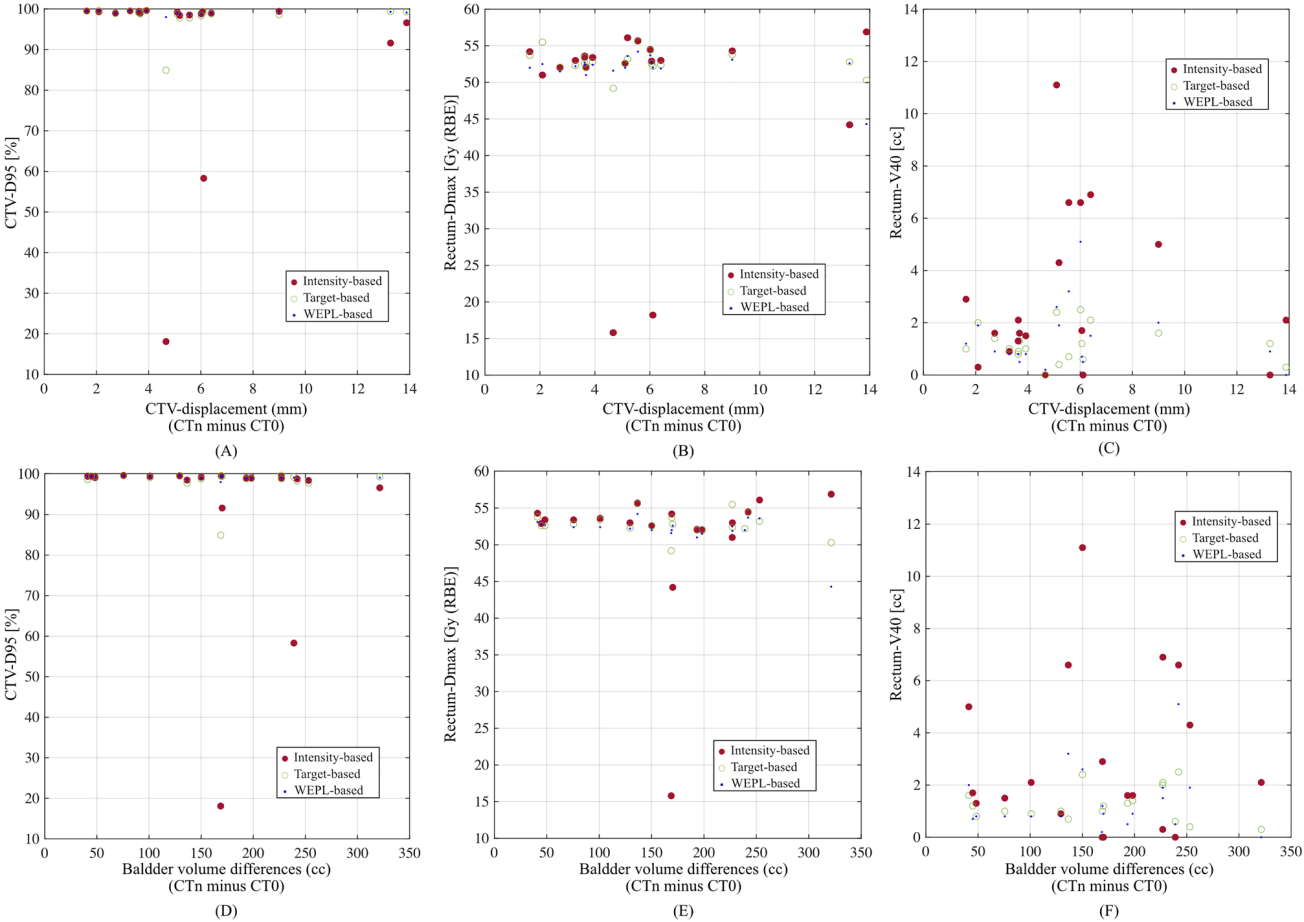
Relationship between CTV displacement and dose metrics, (**A**) CTV-D95, (**B**) rectum-Dmax, (**C**) rectum-V40. Relationship between bladder volume differences (CTn minus CT0) and dose metrics, (**D**) CTV-D95, (**E**) rectum-Dmax, (**F**) rectum-V40. This image is created by MATLAB R2021a, (Mathworks, Natick MA, USA, https://www.mathworks.com).

Figure 6 shows the relationship of CTV displacement and bladder volume, with dose metrics (CTV-D95, rectum-Dmax, and rectum-V40), showing that these results were not related to the dose metrics. To evaluate correlation quantitatively, Pearson correlation coefficients for respective image registrations were summarized in Table 1. For the relationship of CTV displacement to the rectum dose metrics (V40, V30 and V20) was moderate correlations (= 0.23 to 0.35) for all image registrations. Correlation value for the WEPL-based image registration (= 0.31) was higher than those for other registrations, however, it was weak correlation. While there was no correlation between the bladder volume difference and dose metrics (CTV-D95: −0.04 to 0.13, rectum V40, V30 and V20: −0.16 to 0.01).

**Table 1:** Pearson correlation coefficients of the relationship between dose metrics and CTV displacement/bladder volume differences for respective image registrations.

		CTV	Rectum			
		D95 (%)	Dmax [Gy(RBE)]	V40 (cc)	V30 (cc)	V20 (cc)
CTV-displacement (mm)	Intensity-based	0.05	0.07	0.25	0.23	0.23
	Target-based	0.13	0.11	0.35	0.33	0.33
	WEPL-based	0.31	0.05	0.29	0.29	0.26
Bladder volume differences (cc)	Intensity-based	0.05	0.01	−0.05	−0.05	−0.05
	Target-based	−0.04	−0.08	−0.14	−0.15	−0.16
	WEPL-based	0.13	−0.12	−0.03	−0.02	−0.02

**Figure 7:**
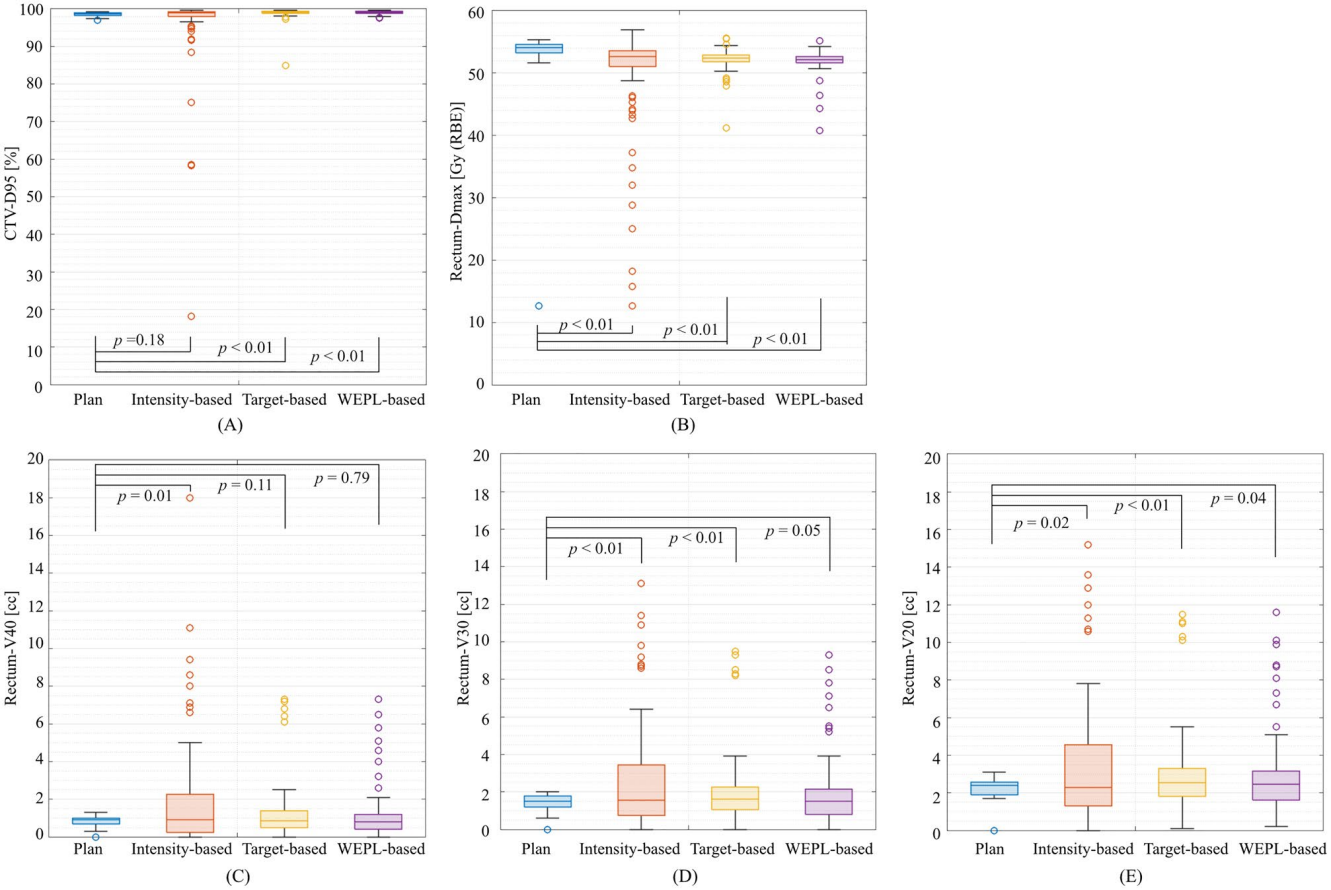
Boxplot of the dose metrics on the planning CT, the treatment CTs with the intensity-based image registration, target-based image registration, and WEPL-based image registration for respective fractions. (**A**) CTV-D95. (**B**) Rectum-Dmax. (**C**) Rectum-V40. (**D**) Rectum-V30. (**E**) Rectum-V20. The central horizontal line in the box indicates the median, and the bottom and top edges of the box indicate the 25th percentile (q1) and 75th percentile (q3), respectively. The whiskers extend to the most extreme data points not considered outliers. An outlier (marked as a circle) is identified if it is > q3 + (q3 − q1) × 1.5 or < q1 − (q3 − q1) × 1.5. This image is created by MATLAB R2021a, (Mathworks, Natick MA, USA, https://www.mathworks.com).

Dose assessment metrics averaged over all patients on the planning CT and the treatment CTs are summarized in Fig. 7 and Table 2. For the CTV coverage, the D95 values on the planning CT were > 95% of the prescribed dose in all cases. The mean CTV-D95 values were 95.8 ± 11.5% and 98.8 ± 1.7% with intensity-based image registration and target-based image registration, respectively. The intensity-based image registration resulted in seven cases receiving less than 95% of the CTV-D95; the target-based image registration decreased the number of such cases to one. The WEPL-based image registration showed 99.0 ± 0.4% of cases receiving CTV-D95, with no case receiving less than the D95. CTV-D95 value with the intensity-based image registration (*p* = 0.18) was statistically degraded from that for the planning CT. Regarding to the target-based and the WEPL-based image registration, the CTV-D95 values for the treatment CT were improved to be close to that for the planning CT (*p* < 0.01 for both image registrations).

**Table 2:** Summary of the dose assessment averaged over all patients.

		Planning CT									Treatment CT									
						Intensity-based image registration					Target-based image registration					WEPL-based image registration				
		Mean	SD	Min	Max	Mean	SD	Min	Max	*p* value	Mean	SD	Min	Max	*p* value	Mean	SD	Min	Max	*p* value
CTV	D95 (%)	98.5	0.6	97.0	99.2	95.8	11.5	18.1	99.7	0.18	98.8	1.7	84.9	99.6	0.00	99.0	0.4	97.5	99.6	0.00
Rectum	Dmax [Gy (RBE)]	52.4	8.2	12.7	55.3	49.4	9.1	12.7	56.9	0.00	52.2	1.8	41.2	55.6	0.00	51.9	1.9	40.8	55.2	0.00
	V40 (cc)	0.8	0.3	0.0	1.3	2.0	3.1	0.0	18.0	0.01	1.3	1.6	0.0	7.3	0.11	1.2	1.5	0.0	7.3	0.79
	V30 (cc)	1.4	0.4	0.0	2.0	2.8	3.7	0.0	20.9	0.00	2.1	2.0	0.0	9.5	0.00	2.0	1.9	0.0	9.3	0.05
	V20 (cc)	2.3	0.6	0.0	3.1	3.7	4.3	0.0	24.3	0.02	3.0	2.3	0.1	11.5	0.00	3.0	2.4	0.2	11.6	0.04

Since the WEPL-based image registration included the rectal and CTV WEPL penalty, the WEPL-based image registration reduced the rectal-Dmax to 51.9 ± 1.9% from that of 52.2 ± 1.8% with the target-based image registration. While rectum doses (V20, V30 and V40) for the WEPL-based image registration (3.0 ± 2.4 cc, 2.0 ± 1.9 cc and 1.2 ± 1.5 cc) were a bit smaller than those for the target-based image registration (3.0 ± 2.3 cc, 2.1 ± 2.0 cc and 1.3 ± 1.6 cc).

### Computation time

Computation time for DIR averaged over all patients was 42.1 ± 0.3 s per CT data set.

Computation time for the intensity-based and WEPL-based image registrations averaged over all patients were 0.08 ± 0.03 s and 11.0 ± 4.3 s, respectively. While computation time for the target-based image registration was negligibly small because of just subtracting the center of mass of the plan-PTV and treatment-CTV. Since the target-based and the WEPL-based image registrations required DIR, total image registration time was 0.08 ± 0.03 s for the intensity-based image registration, 42.1 s for the target-based image registration, and 53.1 s for the WEPL-based image registration.

## Discussion

We developed WEPL-based image registration and compared it with intensity-based image registration and target-based image registration using serial prostate CT data. The WEPL-based image registration prevented dose degradation and minimized rectal dose due to interfractional variation better than other two image registrations. we found the WEPL-based image registration improved CTV-D95 more than 97.5% over intensity-based image registration (> 18.1%) and target-based image registration (> 84.9%) in our study. The number of actual treatment fractions was 12 in our treatment protocol; however, our results with weekly serial CTs provided useful information, which closely reflects the clinical situation. Medical staff always endeavor to minimize the magnitude of interfractional variation; however, it is impossible to remove completely. Image registration based on a full dose re-calculation i.e. to minimize the difference in dose between treatment and planned CT is typically not practical or available. Images registration based on WEPL is a practical solution that leads to better target dose coverage and reduced OAR dose.

### Image registration

Currently, our prostate treatment protocol does not use CT-CT image registration for patient setup verification, but registers bony anatomical structures to the reference position on digitally reconstructed radiographic (DRR) images, which are derived from planning CT data, using 2D–3D rigid registration software for the acquisition of a pair of x-ray images^33^. When the implanted marker position was located > 5 mm from the markers on DRR, the patient couch was readjusted. It is, therefore, close to a combination of intensity-based and target-based image registration. Although the 2D–3D image registration cannot consider WEPL variations, our results showed a sufficient dose to the target in most cases. A few cases had degraded target coverage, indicating that medical staff should check each patient before irradiation.

The current CT–CT image registration algorithms can be classified as either feature-based or intensity-based^34^. Our proposed method has two new features: using WEPL values as the registration metric and the penalty, which measures the CTV out of the irradiation field, and factors it into the registration function.

The most problematic situation is that the target was not within the beam field (target miss), it can result in under-dosage of the target and over-dosage of the surround tissues. To prevent the under-dosage of the target, we integrated CTV penalty to include the treatment-CTV within the planning-PTV. Moreover, to prevent the over-dosage of the OAR (rectum), the rectum penalty was integrated to keep the rectum and the beam field (the planning PTV) away from each other. While our results show the WEPL difference with the intensity-based image registration was smaller than those with others, the WEPL image registration optimized the above three metrics to provide a better dose distribution for treatment than the other image registrations.

Maeda et al. reported that it is effective to maintain the dose constraint to the rectum and the dose coverage of the prostate with repositioning by manual correction after conventional bone matching^28^. Our study arrived at the same conclusion without manual correction, independent of user skill.

### Dose assessment using CTV

Other studies use PTV for interfractional dose assessment^23,25,35^; however, we evaluated target coverage using CTV. The International Commission on Radiation Units & Measurements (ICRU) Report 78 defines internal margins to compensate for internal uncertainties such as physiologic movement and variations in size, shape, and position of the CTV and uncertainties in factors external to the patient such as setup error^36^. PTV is then calculated by adding the internal margin and setup margin to the CTV. By doing this, the PTV is useful in avoiding targeting inaccuracy during treatment to ensure a sufficient dose to the CTV.

### Interfractional variation

Interfractional CTV/organ positional variations were observed and affected the dose distribution, however, we evaluated the relationship between interfractional positional/volume variations and dose distributions for 80 fractions (= 20 patients × 4 treatment CTs) and did not find statistically significant differences. Russo et al*.* evaluated interfractional dose distribution using serial cone-beam CT with bony structure registration, and concluded that dose variations due to rectal and bladder volume variations could be improved by image guidance^35^. However, interfractional change resulted in degraded target coverage for some fractions.

Recently, we started clinical trials of hypofractionated prostate treatment using four fractions. A shorter treatment course avoids some interfractional tumor/organ shape variations, but interfractional target positional variation cannot be avoided. As hypofractionated treatment increases the prescribed dose per fraction, it is important to optimize the target coverage and minimize the rectal dose.

We added 10-mm PTV margins except to the posterior aspect of the prostate CTV. This does not mean interfractional positional error would be acceptable within this PTV margin, because this PTV margin does not include beam range. Also, lateral beam fields in the prostate treatment pass through high-density tissues such as the femurs and iliac bones, which would affect the dose distribution more than attenuation by soft tissue. If the margin including WEPL variation were added to the CTV (beam-specific target volume)^37^, the positional variation may be acceptable.

### Clinical implementation

The intensity-based image registration and the target-based image registration have been integrated into the commercially available treatment system. These image registrations were performed automatically and manually respectively. When fiducial markers were implanted into the prostate, target-based image registration was easier to perform. However, the target-based image registration without fiducial markers and the WEPL-based image registration required CTV and rectum contours on the treatment-CT. These VOI counters can be obtained by manual delineation or applying DIR with the treatment CT and the planning CT. Since currently automatic delineation with DIR is often preferred, there is no additional process for the clinical staff. Moreover, the WEPL-based image registration required independent registration for respective beams. However, this situation is often observed in carbon-ion beam treatment center used fixed beam ports system. A treatment couch was rotated to increase beam angle selection and patient setup was performed for respective beams.

Because WEPL-based image registration took approximately 53.1 s, it does not significantly impact treatment throughput.

Since the target-based image registration aligned the target positions, it is useful for the clinical staff that the target-based image registration provided intuitive visual inspection of the target location before and after registration. While the WEPL-based image registration prevented dose degradation due to interfractional change, however, it does not always provide intuitive visual inspection because aligned patient position by using WEPL information. The target-based image registration, therefore, seems much easier for clinical use. One solution is that visualize WEPL difference map and/or weekly dose distribution might be useful.

### Limitations of this study

This study had some limitations. First, other studies acquired the treatment CT in the treatment room using a gantry mounted CBCT or CT on rails. The advantage of this is that data are acquired in or closer to the treatment position. We acquired treatment CTs in the simulation room in this study. The same procedures (immobilization devices, rectum filling control) were applied to the patients before CT imaging, but the patient setup procedure was not performed before CT imaging. Therefore, significant positional change of the prostate would increase in between the simulation and treatment rooms. If interfractional variation was not clinically acceptable, the patient setup procedure was cancelled and/or retried. The benefit of the WEPL-based image registration might be reduced. For clinical use of the CT-CT image registration, the treatment CT image acquisition is intended to be performed in the treatment room. Therefore, above prostate positional variation could be minimized. In most cases, a final patient setup was performed by using kV image matching of fiducial markers after CT image registration. However, kV image matching should not be used after WEPL-based image registration.

Second, three types of image registration used rigid registration, therefore, DIR accuracy did not affect on the registration results. However, we evaluated dose distributions for respective fractions, not for doses accumulated over time including DIR due to the limitation of DIR accuracy. While the accumulated dose over time was calculated by warping the treatment CTs on the planning CT using DIR, if there was an error, it is not practical to modify the dose distribution correctly. As the pelvic soft tissues have small contrast differences, DIR accuracy could be degraded and affected on the time-accumulated dose distributions. Therefore, we did not evaluate the doses over time.

Third, we used DIR to transfer the VOIs on the planning CT to the treatment CTs. We checked propagated VOIs on the treatment CTs and modified them manually as needed, making room for interobserver error^38^. When CT-CT image registration for patient setup is generated routinely, these problems would remain.

## Conclusion

We developed a CT–CT image registration algorithm using a WEPL variation (WEPL-based image registration), and compared carbon-ion pencil-beam scanning dose distribution using serial prostate CT data with three different types of CT–CT image registration. The target-based image registration improved CTV coverage more than the intensity-based image registration. However, as the magnitude of interfractional variation increased, target coverage with the target-based image registration decreased. While our WEPL-based image registration showed moderate correlation in between CTV displacement and dose metrics. And it improved the target coverage and reduced rectal dose for all cases rather than the target-based and intensity-based image registration techniques. Even though a few treatment centers were available to evaluate dose assessment using the treatment CT, the WEPL-based image registration would be useful for these centers and can potentially improve treatment throughput.

## Methods

### Patient and CT imaging

A total of 19 patients with tumors of the prostate receiving scanned carbon-ion beam treatment at our hospital were enrolled. The pelvic region was immobilized with a urethane resin cushion (Moldcare^®^, Alcare, Tokyo, Japan) and low-temperature thermoplastic shells (Shell Fitter^®^, Kuraray Co., Ltd., Osaka, Japan). No water restriction was imposed, and the rectum was emptied by the patient's effort or a laxative or enema. Two or three fiducial markers were implanted into the prostate.

Weekly serial CT scans (repeat CTs: Weeks 1–4) were acquired under breath-hold conditions at exhalation using a 320-detector CT (Aquilion One Vision^®^, Canon Medical Systems, Otawara, Japan) in the simulation room. CT imaging conditions were based on our clinical protocols, with a tube voltage of 120 kV and section thickness of 2.0 mm. X-ray tube current was adjusted by automatic exposure control. An initial CT (CT0) was acquired for treatment planning purposes. After the initial treatment planning scan, weekly CT scans (repeat CTs: Weeks 1–4) were acquired. Weekly CT scans were not performed on the same weekday for respective patients. The elapsed times between the planning CT and weekly CT scans are summarized in Table 3. The study involved human participants and was approved by the Institutional Review Board of our institution (N21-007) and performed in accordance with the Declaration of Helsinki. All patients provided informed consent to participate in this study.

**Table 3:** Elapsed days after planning CT (CT0).

	CT1	CT2	CT3	CT4
Mean	13.8	18.1	23.3	27.6
SD	3.2	3.5	2.8	2.4
Min	10	15	21	24
Max	24	29	30	33

### Volume of interest (VOI) definition

**Figure 8:**
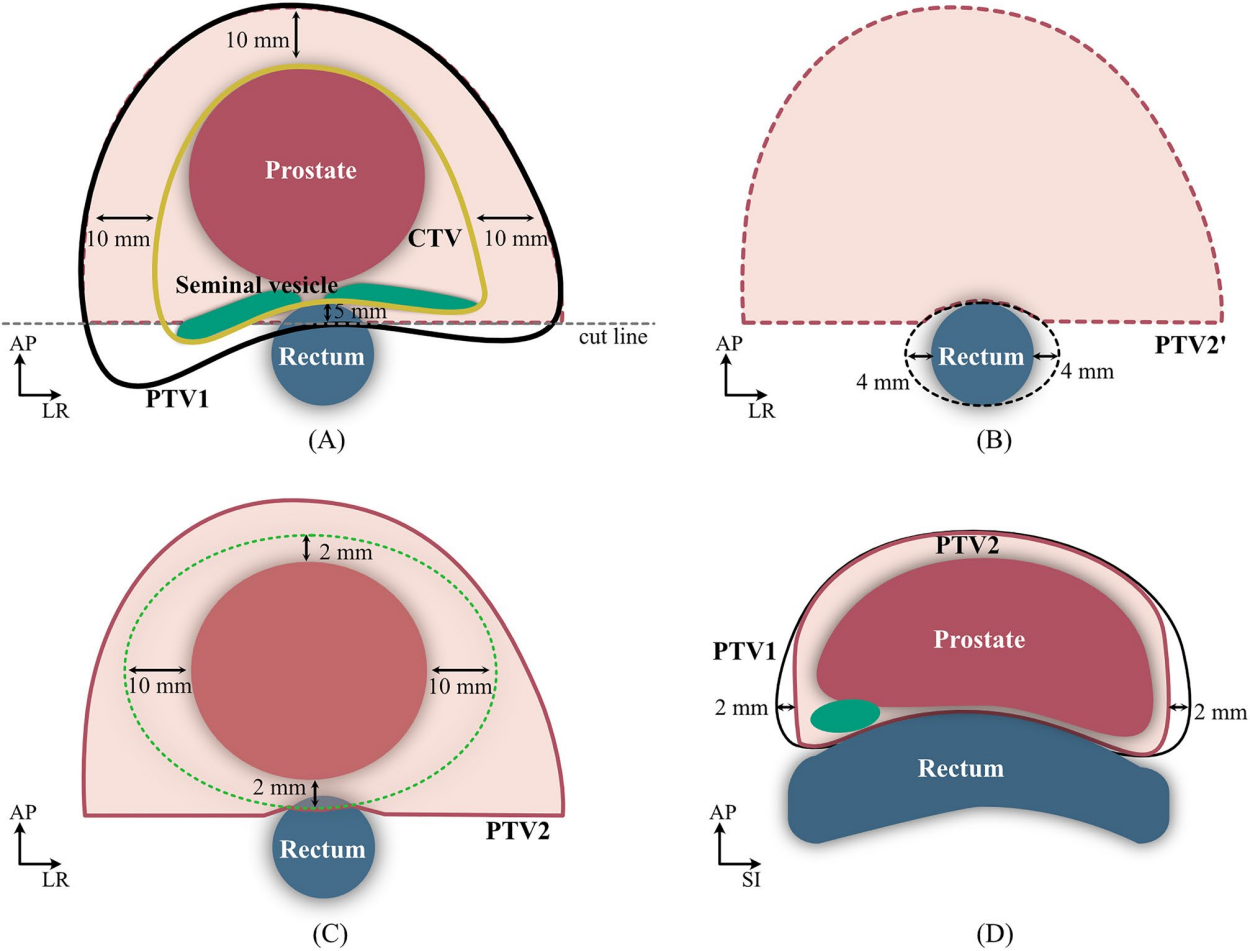
Schematic drawing of the VOI. (**A**) The CTV (yellow line) included the prostate and seminal vesicles. Initial PTV (PTV1) was defined by adding a 10-mm margin to the anterior, lateral, and superior-inferior sides and a 5-mm margin to the posterior side of the CTV. The second PTV (PTV2) was designed as follows: A horizontal line (cut line) was set 5 mm posterior to the CTV posterior aspect on CT image sections. The part of the PTV1 region posterior to the cut line was removed (colored white in (**A**)). (**B**) The rectal ROI was expanded by 4 mm from each lateral border (colored white in (**B**)) and this volume was removed (PTV2'). (**C**) PTV2 was calculated by expanding the prostate region by 10 mm lateral expansion, 2 mm ant/post expansion, forming an approximate semicircle with max diameter at the anterior portion of the rectum. The rectum itself is cropped from the PTV2 and (**D**) deleted 2 mm in inferior and superior sides. This image is created by Affinity Designer version2 (Affinity, Nottingham, England, https://affinity.serif.com/en-us/designer/).

Prostate, seminal vesicles, rectum, and bladder were manually delineated by certified radiation oncologists on the planning CT (CT0). The CTV included the prostate and seminal vesicles (Fig. 8A). The initial PTV (PTV1) was defined by adding a 10-mm margin to the anterior and lateral aspects and a 5-mm margin to the posterior aspect of the CTV.

The second PTV (PTV2) was designed as follows: A horizontal line (cut line) was set 5 mm from the posterior aspect of the CTV on CT images. It is the region above the horizontal line that is colored light pink (Fig. 8A). The rectum was expanded 4 mm on laterally (both sides) and the two 4 mm-thick areas were removed (PTV2') (Fig. 8B). Finally, PTV2 was calculated by expanding the prostate region by 10 mm lateral expansion, 2 mm ant/post expansion, forming an approximate semicircle with max diameter at the anterior portion of the rectum. The rectum itself is cropped from the PTV2 (Fig. 8C). PTV2 was calculated by reducing rectum VOI from PTV2’ and deleted 2 mm in inferior and superior side (Fig. 8D). The PTV design included many steps, however, it can be created in a systematic way and used in our clinical treatment to reduce rectum dose. A ring-shaped VOI between 2 and 6 mm away from PTV1 was created (Ring-PTV1)^37^.

A 2-mm reduced rectum VOI was calculated to dose distribution (Rectum-2 mm).

All VOIs on the weekly CT images were then automatically calculated from the VOIs on the planning CT using DIR employing B-spline technique with our in-house image registration software^39^. The DIR was performed on Windows 10 environment and is installed on a workstation (Intel^®^ Core i7 CPU@3.7 GHz, 64 GB physical memory) and a single GPU on a board (NVIDIA GTX1080Ti ©, NVIDIA Corporation, Santa Clara, CA, USA), which is equipped with 3584 compute unified device architecture (CUDA) cores, and has 11 GB of memory. Then oncologist and medical physicist checked all VOIs on the weekly CT and modified them, if necessary. Especially, we modified prostate and seminal vesicles in several cases.

To distinguish VOIs on the plan and the treatment CTs, we use “plan-” and “treatment-” such as “plan-CTV”, “plan-PTV” and “treatment-CTV”.

### Image registration

We describe the three types of image registration algorithms, which were based on the rigid image registration. And we compared to register the treatment CT to the planning CT below.

All image registrations were programmed using software developed using the C++ program language (Microsoft Visual Studio 2012^®^, Microsoft, Redmond WA, USA). It works under a Windows 10 environment and is installed on a workstation (Intel^®^ Core i7 CPU3.7 GHz, 64 GB physical memory) and a single GPU on a board (NVIDIA TitanV©, NVIDIA Corporation, Santa Clara, CA, USA), which is equipped with 5120 CUDA cores, and has 12 GB of memory.

#### Intensity-based image registration

In general, image registration is performed by aligning one image to the other such that the differences between the two images are minimized. The most common intensity-based image registration minimizes the sum of the squared difference of plan and treatment CT images^40–43^. This metric requires minimal computation power and is intuitively understandable for registering two images.

The alignment value was calculated in translations and rotations by

1 $$\Delta V=\mathrm{arg}\underset{\Delta V}{\mathrm{min}}E\left(\Delta V\right),$$

where $\Delta V$ is an alignment vector that denotes CT position and rotation in the treatment room.

$E\left(\Delta V\right)$ was defined as

2 $$E\left(\Delta V\right)={\Sigma }_{i\in\Omega }{\left|{T}_{i}\left({V}_{p}+\Delta V\right)-{P}_{i}\left({V}_{p}\right)\right|}^{2},$$

where ${T}_{i}\left({V}_{p}\right)$ and ${P}_{i}\left({V}_{p}\right)$ were treatment and planning CT images respectively, which were set at the at the position ${V}_{p}$ determined at the planning; ***i*** is a 3D vector which denotes a point in the room; $\Omega$ is a set of calculation points, which was assigned within the body.

#### Target-based image registration 

In the first step, target-based image registration aligns the treatment-CTV to the plan-CTV. The translation elements of $\Delta V$ were simply calculated by

3 $$\Delta V={{\varvec{g}}}_{p}-{{\varvec{g}}}_{t},$$

where ${{\varvec{g}}}_{p}$ is the center of mass of the plan-CTV.${{\varvec{g}}}_{p}$ is calculated by

4 $${g}_{p}=\frac{1}{N\left({\Omega }_{p,{V}_{p}}\right)}{\Sigma }_{{\Omega }_{p,{V}_{p}}}{\varvec{i}},$$

where ***i*** is the 3D position in the room; $V$ is a six-dimensional vector that denotes CT position and rotation in the treatment room; ${\Omega }_{p,{V}_{p}}$ is the set of 3D calculation points inside the plan-CTV when the planning CT position is ${V}_{p}$; $N\left({\Omega }_{p,{V}_{p}}\right)$ derives the number of voxels within the ${\Omega }_{p,{V}_{p}}$. ${{\varvec{g}}}_{t}$ is the center of mass of the CTV on the treatment CT, calculated by

5 $${g}_{t}=\frac{1}{N\left({\Omega }_{t,{V}_{t}}\right)}{\Sigma }_{{\Omega }_{t,{V}_{t}}}i,$$

where ${\Omega }_{t,{V}_{t}}$ is the set of 3D calculation points inside the treatment-CTV when the treatment CT position is ${V}_{t}$.

Note that the rotation value of $\Delta V$ calculated by target-based image registration is 0, which means that treatment CT moved only by translation.

#### WEPL-based image registration

Since WEPL is related to the charged particle beam stopping position, WEPL analysis is effective for measuring the range of variations affecting the penetration of charged particle beams. Using WEPL analysis for each beam, WEPL-based image registration registered treatment CT to the plan CT by minimizing the WEPL differences over the CTV and OAR. This metric was applied to our image registration algorithm. WEPL is calculated by the physical length (*d*) to the radiological water equivalent pathlength (*d'*) using the following equation:

6 $$WEPL=\sum_{i}\left(\Delta {d}_{i}\right)\cdot {\rho }_{i},$$

where ***i*** is an iterator over the points for the ray path of the treatment beam; ∆*d*_i_ is the pathlength in tissue with the relative stopping power *ρ*_i_. The value *ρ*_i_ is calculated from the transformed CT image pixel values by a conversion table using polybinary calibration methods^44^.

To minimize the range variations, each pixel value of the CT images is changed into WEPL values and the alignment values are calculated in the same manner as the intensity-based image registration algorithm.

**Figure 9:**
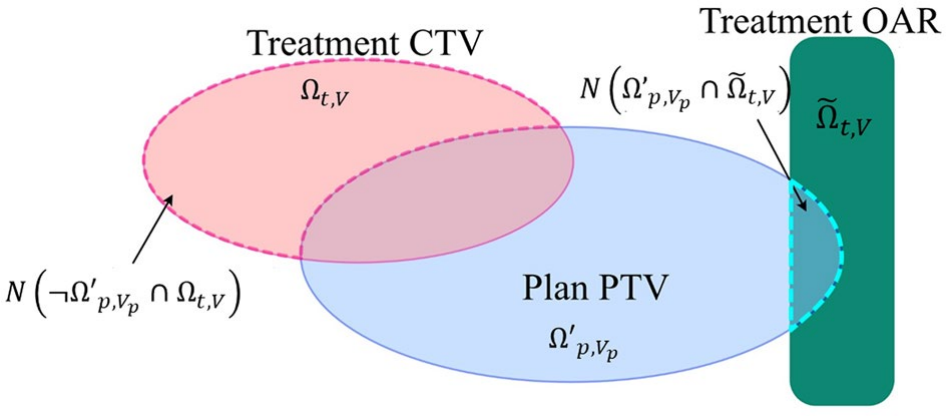
Schematic drawing of the penalty function: $f\left(V+\Delta V\right)$ is the CTV on the treatment CT extending from the PTV on the planning CT. ${f}_{r}\left(V+\Delta V\right)$ is the OAR on the treatment CT overlapped with the plan PTV. This image is created by Affinity Designer version2 (Affinity, Nottingham, England, https://affinity.serif.com/en-us/designer/).

Depending on the patient position at the time of treatment, the center of mass of the treatment CTV may not be within the irradiation field defined by the plan-PTV before the patient setup procedure. When the treatment CTV is not within the beam field as defined by the planning PTV, the optimizer might not find the global minimum. To avoid this problem, we designed a registration error function $E\left(\Delta V\right)$ involving a penalty function which returns the volume of the CTV’s center of mass extending from the plan-PTV (Fig. 9). The function was defined as the following;

7 $$E\left(\Delta V\right)={\Sigma }_{i\in\Omega }{\left|{T}_{i }\left(V+\Delta V\right)-{P}_{i}\left({V}_{p}\right)\right|}^{2}+f\left(V+\Delta V\right)+\lambda {\left|\Delta V\right|}^{2},$$

where ${T}_{i}\left(V\right) \mathrm{and} {P}_{i}\left({V}_{p}\right)$ are WEPL values transformed from treatment and planning CT images for each voxel by using Eq. (6), whose position in the simulation room is represented by $V$ and ${V}_{p}$, which is the position determined on the planning CT; $\Delta V$ is an alignment vector that denotes CT position and rotation in the treatment room.; ***i*** is an iterator over the set points in the room; $\Omega$ is a set of calculation points, which is assigned:

8 $$\Omega ={\Omega }_{p,{V}_{p}}\cup {\Omega }_{t,{V}_{t}}\cup {\widetilde{\Omega }}_{p,{V}_{p}}\cup {\widetilde{\Omega }}_{t,{V}_{t},}$$

where ${\widetilde{\Omega }}_{p,{V}_{p}}$ is the set of calculation points in the OAR (e.g., rectum) on the planning CT when the planning CT position is ${V}_{p}$, and ${\widetilde{\Omega }}_{t,{V}_{t}}$ is the set of calculation points in the OAR on the treatment CT when the treatment CT position is ${V}_{t}$.

In Eq. (7), the first term on the right hand was the WEPL difference and $f(V)$ is the penalty function, which returns the sum of the number of the treatment-CTV voxels outside the plan-PTV and the number of the voxels in the OAR on the treatment CT over the plan-PTV as below;

9 $$f\left(V\right)={\eta }_{1}N\left(\neg {\mathrm{\Omega {^{\prime}}}}_{p,{V}_{p}}\cap {\Omega }_{t,{V}_{t}}\right)+{\eta }_{2}N\left({\mathrm{\Omega {^{\prime}}}}_{p,{V}_{p}}\cap \widetilde{\Omega }{ }_{t,{V}_{t}}\right),$$

where ${\Omega {^{\prime}}}_{p,{V}_{p}}$ is the set of calculation points in the PTV on the planning CT when the planning CT position is ${V}_{p}$ and the hyper-parameters ${\eta }_{1}$ and ${\eta }_{2}$ were set at 1000 and 1000, respectively.

In Eq. (7) the third term is a regularizer to avoid the calculation of large alignment values $\Delta V$ and the hyper-parameter $\lambda$ was set at 100,000. We minimized the registration error function by using the Lucas-Kanade algorithm^45^ and obtained:

10 $$\Delta V={H}^{-1}\left({\Sigma }_{i\in\Omega }{\nabla }_{i}\left(V\right)\left({T}_{i}\left(V\right)-{P}_{i}\left({V}_{p}\right)\right)-\frac{1}{2}{\nabla }_{f}\left(V\right)\right).$$

In this expression, ${\nabla }_{i}\left(V\right)={\left(\frac{\partial {T}_{i}\left(V\right)}{\partial {r}_{x}},\frac{\partial {T}_{i}\left(V\right)}{\partial {r}_{y}},\frac{\partial {T}_{i}\left(V\right)}{\partial {r}_{z}},\frac{\partial {T}_{i}\left(V\right)}{\partial {t}_{x}},\frac{\partial {T}_{i}\left(V\right)}{\partial {t}_{y}},\frac{\partial {T}_{i}\left(V\right)}{\partial {t}_{z}}\right)}^{T},$ is the gradient of image ${T}_{i}\left(V\right)$ computed in the 6-degrees of freedom coordinate; ${\nabla }_{f}\left(V\right)$ is the gradient of function $f\left(V\right)$ in the 6-degrees of freedom coordinate; $H={\Sigma }_{i\in\Omega }{\nabla }_{i}\left(V\right){\nabla }_{i}^{T}\left(V\right)+\lambda I$ is the Hessian matrix, where $I$ is the unit matrix.

To minimize Eq. (7) the Lucas-Kanade algorithm initially sets value $V$ as ${V}_{p}$ and then iteratively solves for increments to the parameter $\Delta V$ by calculating Eq. (11) and then the parameter $V$ is updated:

11 $$V\leftarrow V+\Delta V.$$

Since we added the penalty function to minimize excessive dose for the OAR, WEL difference from WEPL-based image registration might not be always lower than that from the intensity-based image registration, target-based image registration. However, WEPL-based image registration could provide better registration position balanced respective conditions.

### Treatment planning

#### Initial dose calculation

Initial dose distributions for carbon ion pencil-beam scanning therapy were calculated using the planning CT data, and the details of treatment planning were previously reported^46^. A prescribed dose of 51.6 Gy (RBE) was administered to the PTV1 with D50%. The number of treatment fractions used was 12. Two different beam angles (90° and 270°) and single field uniform dose were selected. The treatment planning parameters (beam spot position and beam spot weight) were optimized to satisfy the dose constraints using the RBE-weighted absorbed dose^47^ to satisfy the following metrics: PTV2-D95% and CTV-D95% > 95% of the prescribed dose, PTV1 minimum dose was > 43.8 Gy (RBE), and rectal dose was less than the reference dose volume histogram, which was prevented rectal complications derived from our prostate treatment with carbon-ion beam treatment^48,49^. Treatment planning optimization objectives are summarized in Table 4. All dose calculations were calculated with a pencil beam algorithm^50^ and performed on a workstation (RayStation 6.99; RaySearch Laboratories AB, Stockholm, Sweden).

**Table 4:** Optimization objectives.

VOI	Type	Dose	Weight
PTV1	Min dose	43.8 Gy (RBE)	100
PTV2	Uniform dose	51.6 Gy (RBE)	300
	Max dose	54.18 Gy (RBE)	300
	Min dose	49.14 Gy (RBE)	300
Ring-PTV1	Max dose	49.08 Gy (RBE)	30
Rectum-2 mm	Max dose	41.28 Gy (RBE)	250
Rectum	Max DVH	60.00 Gy (RBE) to 4% volume	200
	Max DVH	40.00 Gy (RBE) to 5% volume	200
	Max DVH	30.00 Gy (RBE) to 6% volume	200
	Max DVH	20.00 Gy (RBE) to 6% volume	200
	Max DVH	10.00 Gy (RBE) to 8% volume	200

#### Weekly dose calculation

**Figure 10:**
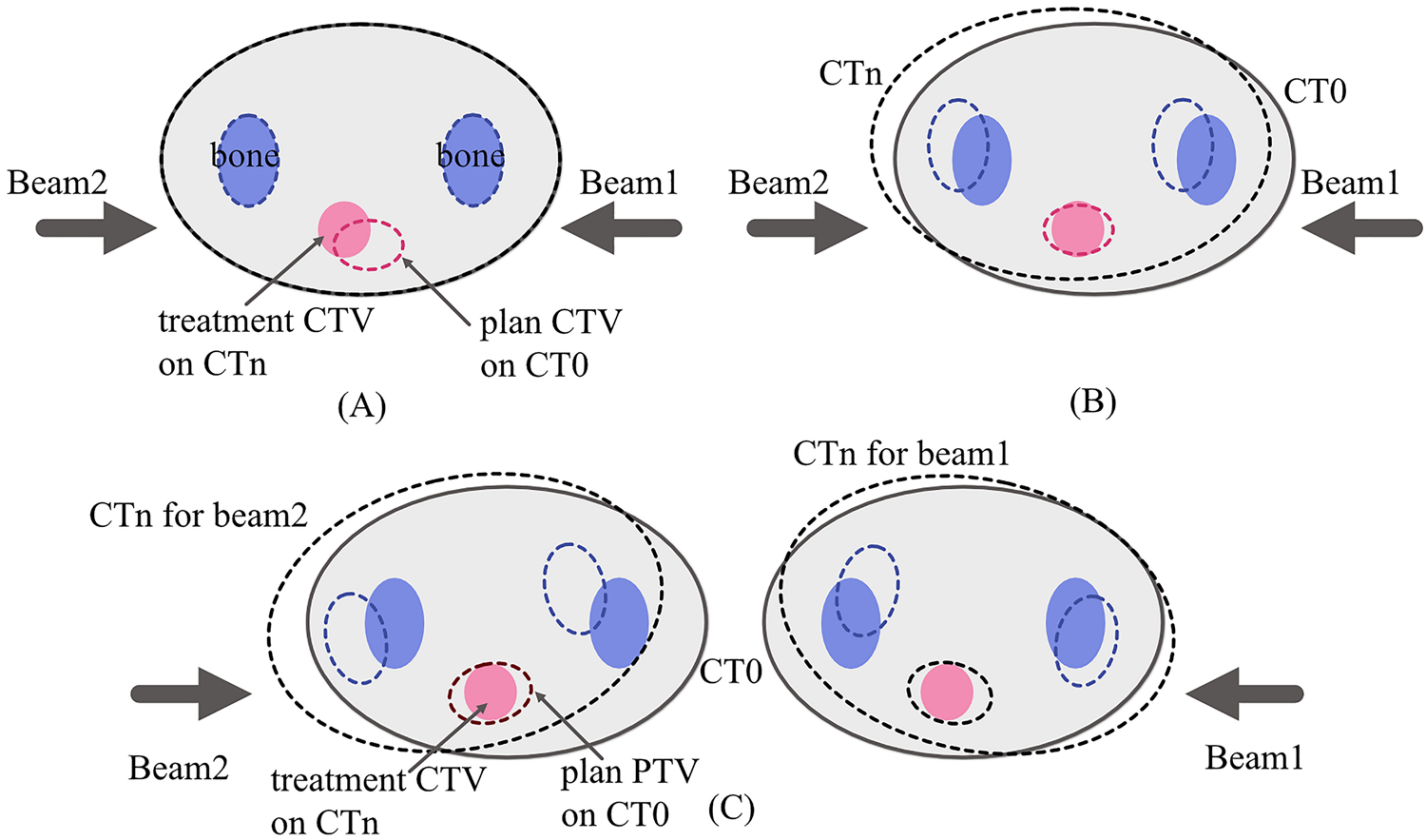
(**A**) Intensity-based image registration designed to minimize the difference between the planning CT (CT0) and the treatment CT (CTn). Dose distributions for beam 1 and beam 2 were calculated on the registered treatment CT. (**B**) Target-based image registration of the treatment CTV isocenter to the planning CTV. Dose distributions were calculated on the registered treatment CT. (**C**) Treatment CTs registered with the WEPL-based image registration for Beam1 and Beam 2. Dose distribution for each beam was calculated by using the treatment CT registered by the WEPL-based method. This image is created by Affinity Designer version2 (Affinity, Nottingham, England, https://affinity.serif.com/en-us/designer/).

Weekly dose distributions were calculated by applying the treatment planning parameters (beam angle, beam weighted spots in respective energy layers) to the treatment CTs (CT1–CT4), which were registered three times using the three different image registration techniques (see “WEPL variation with the image registration”). Since the intensity-based image registration and target-based image registration provided a single registered position for respective beam angles, doses from the two beams were summed for the respective beam angles on the same registered CT data (Fig. 10A,B).

While the WEPL-based image registration gave different registered positions for respective beam angles, the treatment planning parameters on the workstation were defined for each beam angle, and it is impossible to change beam angles with the treatment planning parameters. Dose distributions for the respective beam angles were calculated on the treatment CT data with different registered positions (CTn for beam1 and CTn for beam2, n is integer between 1 and 4) over the four treatments (Fig. 10C). Then to calculate summed dose distribution, the dose distributions for the respective beam angles were registered to the same CT coordinates. Due to the limitations of DIR accuracy, we did not calculate time-accumulated dose distributions throughout the treatment course (CT1–CT4)^51,52^.

### Evaluation

#### CTV displacements

The treatment CT was registered to the planning CT by the rigid bone matching. And then the center of mass of the CTV on the treatment CT (${{\varvec{g}}}_{t}$) was subtracted from that on the planning CT (${{\varvec{g}}}_{c}$), and it was expressed by the Euclidian distance as follows:

12 $$\left|\Delta {V}_{c}\right|=\left|{{\varvec{g}}}_{c}-{{\varvec{g}}}_{t}\right|.$$

#### Volume variations

Volume variations were calculated using prostate and bladder VOIs on the plan and the treatment CTs (CTn minus CT0).

#### WEPL variation with image registration

The accuracy of intensity-based image registration and target-based image registration was generally assessed visually. Since WEPL based image registration did not always produce registrations where anatomical structures matched, visual evaluation is not appropriate. Instead, WEPL variations were compared in between the plan and treatment CTs with respective CT image registration algorithms.

#### Computation time

Before calculating three types of CT image registrations, VOIs on the planning CT should be propagated to the weekly CTs by using DIR. Therefore, the computation time for DIR and the image registration were evaluated.

#### Dose assessment

Dose assessments for respective image registrations were performed using D95% to the CTV, and the percentage of the VOI irradiated with > *n* Gy (RBE) (V*n*) for the rectum (V40, V30, and V20). These metrics were evaluated on the planning CT and the treatment CTs (CT1–CT4). All metrics in the respective plans were compared using the Wilcoxon signed-rank test (MATLAB R2021a®, Mathworks, Natick MA, USA). All *p* values were two-sided and those < 0.05 were regarded as statistically significant. The relationship between CTV displacement and dose metrics were evaluated using Pearson correlation coefficients.

### Ethics statement

The study involved human participants and was approved by the Institutional Review Board of National Institutes for Quantum Science and Technology (N21-007) and performed in accordance with the Declaration of Helsinki. All patients provided informed consent to participate in this study.

## Data Availability

The data that support the findings of this study are available on request from the corresponding author but restrictions apply to the availability of these data, which are not publicly available due to ethical restrictions.

## Abbreviations

CBCT: Cone-beam computed tomography
CT: Computed tomography
CTV: Clinical target volume
DIR: Deformable image registration
DRR: Digitally reconstructed radiographs
DVH: Dose-volume histogram
GTV: Gross tumor volume
IMRT: Intensity modulated radiotherapy
Max: Maximum
Min: Minimum
OAR: Organ at risk
PTV: Planning target volume
RBE: Relative biological effectiveness
SD: Standard deviation
VOI: Volume of interest
WEPL: Water equivalent pathlength
